# Insights for the application of TILs and AR in the treatment of TNBC in routine clinical practice

**DOI:** 10.1038/s41598-020-77043-9

**Published:** 2020-11-18

**Authors:** Agnese Losurdo, Rita De Sanctis, Bethania Fernandes, Rosalba Torrisi, Giovanna Masci, Elisa Agostinetto, Wolfgang Gatzemeier, Valentina Errico, Alberto Testori, Corrado Tinterri, Massimo Roncalli, Armando Santoro

**Affiliations:** 1 Department of Medical Oncology and Hematology, Humanitas Clinical and Research Center - IRCCS, Via Manzoni 56, 20089 Rozzano, Milan Italy; 2 Department of Biomedical Sciences, Humanitas University, Via Rita Levi Montalcini 4, 20090 Pieve Emanuele, Milan Italy; 3 Department of Pathology, Humanitas Clinical and Research Center - IRCCS, via Manzoni 56, 20089 Rozzano, Milan Italy; 4 Department of Breast Surgery, Humanitas Clinical and Research Center - IRCCS, via Manzoni 56, 20089 Rozzano, Milan Italy

**Keywords:** Breast cancer, Oncogenesis

## Abstract

Triple negative breast cancer (TNBC), usually presenting with a very aggressive phenotype, is a heterogeneous entity. We aim to discuss new biomarkers, suitable for prognostic and predictive purposes. We retrospectively collected clinical variables and immunohistochemical characteristics of early TNBCs, specifically focusing on the prognostic and predictive significance of tumor infiltrating lymphocytes (TILs) and androgen receptor (AR) expression, assessing their correlation with clinical variables. Among 159 patients, TILs were significantly higher in younger patients and with lower BMI, and in tumors with higher ki-67 and greater nodal involvement; conversely, AR was significantly higher in older patients and in tumors with lower ki-67. Interestingly and in line with literature, both TILs level and ARs expression were lower within metastatic sites, in patients who developed distant metastases, compared to those found in the primary site. Small (pT1) and node negative tumors were highly represented and no correlation of either TILs or AR with prognosis could be observed. Our findings support the use of stromal TILs to identify a more aggressive, but chemo-sensitive phenotype, mostly represented in younger women, while AR may identify a less aggressive, slow-growing luminal TNBC subtype, more common among older patients. TILs and AR are worth implementing in routine clinical practice to refine prognosis even if, in our case series, we couldn’t identify a significant correlation of the two variables with either disease-free and overall survival.

## Introduction

Breast cancer (BC) is the most common cancer in women and the second cause of cancer female mortality^1^. In routine clinical practice, BC can be classified into prognostic and predictive subtypes based on the immunohistochemical (IHC) expression of the estrogen receptor (ER), the progesterone receptor (PgR) and the human epidermal growth factor receptor type 2 (HER2), with or without in situ hybridization of the latter, in equivocal 2 + tests^2^.

Triple negative (TN) BC accounts for 15–20% of BCs and it is characterized by the absence of ER, PgR and HER2 overexpression; it usually presents with a very aggressive phenotype, frequently exhibiting high Ki-67 index and high histological grade. Due to its intrinsic aggressive clinical behavior and the lack of recognized predictive biomarkers to be used as targets for therapy, patients with TNBC have a poorer outcome compared with those presenting with other BC subtypes. Chemotherapy is still the standard of care for TNBC, in both early (neo-adjuvant and adjuvant) and advanced-stages of the disease, with anthracycline and taxanes based regimens routinely used in the early settings and sequential single-agent chemotherapy, with a predominant role for platinum-derived agents, to be used in the metastatic setting^3–6^.

It is widely accepted that TNBC itself is a heterogeneous entity and many efforts have been made to better refine the understanding of its features and to identify potential biomarkers for targeted therapies to be effectively used in this challenging BC subtype. Stromal tumor infiltrating lymphocytes (TILs) in TNBC, evaluated as a continuous variable, following the recommendations of the International TILs working group^7^, retain an important prognostic significance, with an improvement in both disease-free survival (DFS) and overall survival (OS)^8–11^. Moreover, TILs have been shown to predict pathological complete response (pCR) in early TNBC following neoadjuvant chemotherapy^12–14^. If TILs have been proved to be more represented in the more chemo-sensitive TNBC subtype, the androgen receptor (AR) seems to identify a “luminal”, less aggressive phenotype. AR has been detected in 25–35% of TNBC and AR negativity has been associated with a shorter DFS and OS as compared to AR-positive TNBCs^15–19^. Indeed, the prognostic role of AR in TNBC has been considered controversial, as some authors reported AR expression to be associated to worse outcome^20–22^ and the lack of prospective data concerning the interaction between AR and OS. Nevertheless, three recent large meta-analyses confirmed longer DFS in AR-positive versus AR-negative BC patients^23–25^ and multiple studies (both single and multicenter case series and prospective studies) have shown AR expression to be significantly associated with lower mitotic index and lower tumor grade at diagnosis^15,17,26–28^.

In their pivotal paper, Lehmann et al. defined different TNBC subtypes on the basis of gene-expression profiles and among them they could distinguish an immunomodulatory subgroup and a luminal AR-expressing group^29^. Nevertheless, these gene-expression based subtypes, although carrying prognostic and predictive information, due to their complexity, cannot be routinely integrated into clinical practice. Our study manly focuses on exploring the potential role in daily clinical practice of TNBC treatment for TILs and AR expression, and their correlation with clinical variables.

## Methods

### Study population: clinical variables

We retrospectively collected, in an ad hoc data base, the clinical-pathologic characteristics of early TNBC, consecutively treated at our Institution between 2006 and 2014. Information on clinical history and follow-up was collected from patients’ medical charts. All patients received both the local (conservative or radical surgery, either followed or not by complementary or loco-regional radiotherapy) and systemic treatment with neo-adjuvant and/or adjuvant chemotherapy. Patients were eligible for the study if they had confirmed TNBC at the time of surgery and if data on surgical, radiation and medical treatment with follow-up or last contact information were available.

Body mass index (BMI) was assessed for all patients based on weight and height at the time of primary surgery and calculated as the weight (in kilograms) divided by the square of the body height (in meters), expressed in units of kg/m^2^.

Neutrophils-to-lymphocytes ratio (NLR) was calculated as the absolute neutrophil count divided by the absolute lymphocyte count based on preoperative blood values (5 to 1 day before surgery).

All procedures were conducted in accordance with the Declaration of Helsinki and have been approved by the local ethics committee (Independent Ethical Committee IRCCS Istituto Clinico Humanitas, protocol number ONC/OSS-05/2017). Written informed consent to the use of clinical data for scientific purposes had been provided by all patients at the time of access for surgery.

### Immunohistochemical analysis

Immunohistochemical analysis on BC specimens were performed at our Pathology Department and tumors were defined as TNBC if ER (Ventana, Clone SP1, pre-diluted) and PgR (clone 30-9; Ventana Medical Systems Inc, pre-diluted) IHC staining was ≤ 1%, together with 0/1 + score HER2 on IHC (clone 4B5; Ventana Medical Systems Inc, pre-diluted) and/or non-amplified on fluorescent in situ hybridization (FISH) for 2 + score HER2 cases. For each study patient, formalin-fixed paraffin-embedded (FFPE) tumor samples were retrieved from Institutional Pathology archives. Stromal TILs were assessed according to consensus guidelines^7,30^ by one investigator (BF) who was blinded to clinical data. AR expression was measured by IHC, using an anti-androgen receptor (clone SP107; Ventana Medical Systems Inc, pre-diluted) and the percentage of AR-positive nuclei was quantified. A cutoff of 10% or greater was used to define an AR-positive tumor^31^. The IHC threshold (= 0 + or < 10% or ≥ 1 + and ≥ 10%) was used for AR.

### Statistical considerations

According to the recommendations of the International TILs working group 2014, we analyzed TILs as a continuous variable. Correlation analysis between TILs, ARs and clinic-pathological data was performed by Spearman test. Differences between groups were calculated by Kruskal–Wallis test.

Comparison of TILs and AR within the primary tumor and their respective metastatic sites was performed by Wilcoxon matched pairs test.

Disease-Free Survival (DFS) was defined as the the time from diagnosis to relapse at any site (local, contralateral or distant). Overall survival (OS) was defined as the time from diagnosis to death from any cause. The Kaplan–Meier method was used to estimate survival curves, the log-rank test was used to test difference between groups. All reported *p*-values are two-sided, and significance level was set at *p* < 0.05.

Statistical analyses were performed using STATA software (version 15).

### Ethical approval

All procedures performed in studies involving human participants were in accordance with the ethical standards of the institutional and/or national research committee and with the 1964 Helsinki declaration and its later amendments or comparable ethical standards. This article does not contain any studies with animals performed by any of the authors.

### Informed consent

Informed consent was obtained from all individual participants included in the study.

## Results

### Patients characteristics

Between 2006 and 2014, 159 patients with TNBC were treated at our Institution; patients’ clinical-pathologic characteristics are summarized in Table 1. Median age was 54 years (range 28–81 years). Most patients (108, 67.9%) received conservative surgery and complementary radiotherapy and, as expected, around 90% of tumors were classified as infiltrating ductal carcinoma. Interestingly, half of patients presented with pT1 tumors and specifically 55 (34.6%) patients had pT1N0 tumors, while 23 (14.5%) patients had pT2N0 tumors, 2 patients pT3N0 (1.3%) and 75 (47.2%) exhibited lymph node involvement (pN1-3). Most of the patients received adjuvant (132, 83%) or neo-adjuvant (9, 5.6%) chemotherapy; in around half of the cases anthracycline and taxane based regimens were used, while 29 (18.2%) patients received non-anthracycline based regimens (CMF or taxane-only regimens). 15 (9.4%) surgically treated patients did not undergo any adjuvant chemotherapy due to concurrent comorbidities or specific patient refusal.

**Table 1:** Patients’ characteristics.

Characteristic	N	%
All pts	159	
Median age, years (range)	54 (29–81)	
**Surgery type**		
Lumpectomy	108	68
Mastectomy	51	32
**Histology**		
IDC	146	92
ILC	5	3
Other	8	5
**pT**		
1	82	52
2	60	38
3	16	10
4	1	1
**pN**		
0	80	50
1	49	31
2	14	9
3	12	8
x	4	3
**NLR**		
0.5–1	9	6
1–1.5	19	12
1.5–2	26	16
2–2.5	37	23
> 2.5	68	43
**TILs**		
0	24	15
1–10	59	37
> 10	68	43
LPBC	5	3
NE	8	5
**AR**		
POS	77	48
NEG	74	47
NE	8	5
**BMI**		
≤ 25	76	48
25–29	27	17
≥ 30	19	12
NE	37	23
**Adjuvant CT**		
Anthracycline-based	20	13
Taxane-based	7	4
Anthracycline/Taxane-based	87	55
CMF	18	11
None	21	13
UK	6	4
**Adjuvant RT**		
Yes	112	70
No	41	26
UK	6	4
**Recurrence**		
Ipsilateral	2	1
Contralateral	4	3
Metastatic	38	24
None	115	72

*IDC* invasive ductal carcinoma, *ILC* invasive lobular carcinoma, *NLR* neutrophils to lymphocytes ratio, *TILs* tumor infiltrating lymphocytes, *LPBC* lymphocyte predominant breast cancer, *NE* not evaluable, *AR* androgen receptor, *POS* positive, *NEG* negative, *BMI* Body Mass Index, *CT* chemotherapy, *CMF* cyclophosphamide-methotrexate-fluorouracil, *UK* unknown, *RT* radiotherapy.

Among 159 tumor specimens analyzed, TILs and AR were evaluable on 151 (94.9%). Median TILs score was 10% (interquartile range 3%-20%). When using 10%, the median stromal TILs score, as our internal cut-off, we could distinguish “low” (71, 47%) to “high” (80, 53%) cases and among the latter category, we were able to identify 5 (3.3%) so called lymphocyte predominant breast cancers (LPBCs), where mononuclear immune cells were more abundant than cancer cells in the stroma inside the tumor burden. Concerning AR IHC staining, we identified 77 (51%) tumor specimens with positive AR staining and 74 (49%) cases with a negative AR staining.

### Correlation of TILs and AR with routine pathologic features

**Figure 1:**
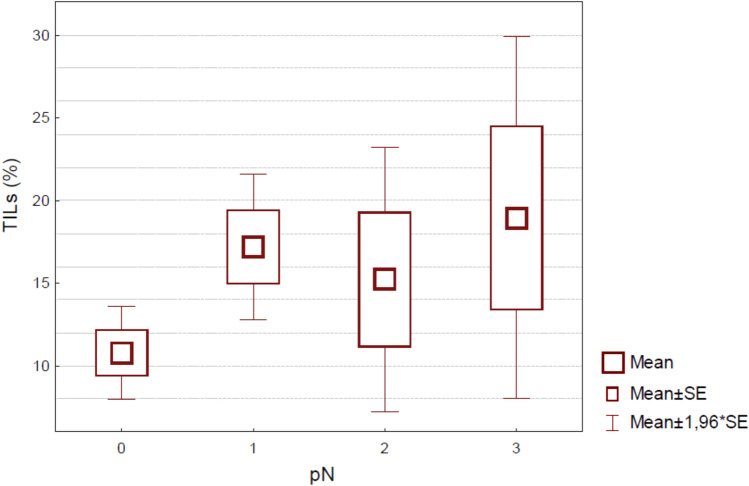
Boxplot by Group. For each TILs value (continue scale) is shown the correspondent extent of lymph node involvement.

We performed a correlation analysis between both TILs and AR and other routinely used pathologic features, such as tumor size, nodal status, histological grade, lympho-vascular invasion (LVI) and Ki-67, and clinical characteristics, such as age, BMI and NLR. TILs were significantly higher in younger patients (Spearman rho − 0.18; p = 0.02) and in patients with lower BMI (Spearman rho − 0.19; p = 0.04), in tumors with higher ki-67 (Spearman rho 0.34; p < 0.001) and greater nodal involvement (p = 0.02; Fig. 1). Post-hoc comparison showed a significant difference between pN0 and pN1 patients, the latter being significantly richer in TILs (data not shown). There was no evidence of association between TILs and size, NLR, grading, pT, histology, LVI, and AR.

**Figure 2:**
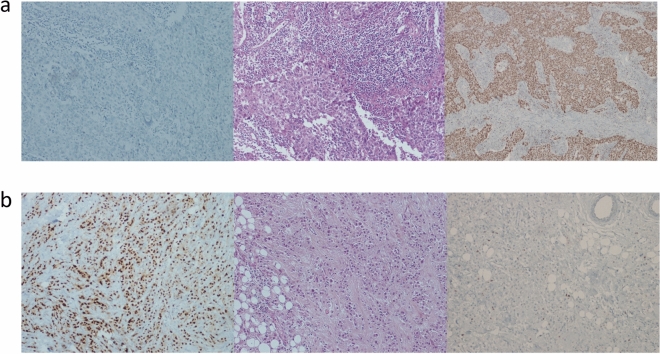
(**a**,**b**). Illustrative IHC slides representing from the left to the right, AR staining, H&E for stromal TILs evaluation and Ki-67 staining. High stromal TILs are associated with higher Ki-67 (**a**), while high AR positivity is associated with lower proliferation index (**b**).

Conversely, AR was found to be significantly higher in older patients (Spearman rho 0.27; p < 0.001), and in tumors with lower ki-67 (Spearman rho − 0.41; p < 0.001). There was no evidence of association between ARs and size, NLR, BMI, grading, pT, pN, histology, and LVI. In Fig. 2 we show representative cases for the relationship between TILs, AR and Ki-67.

### DFS and OS analysis

**Figure 3:**
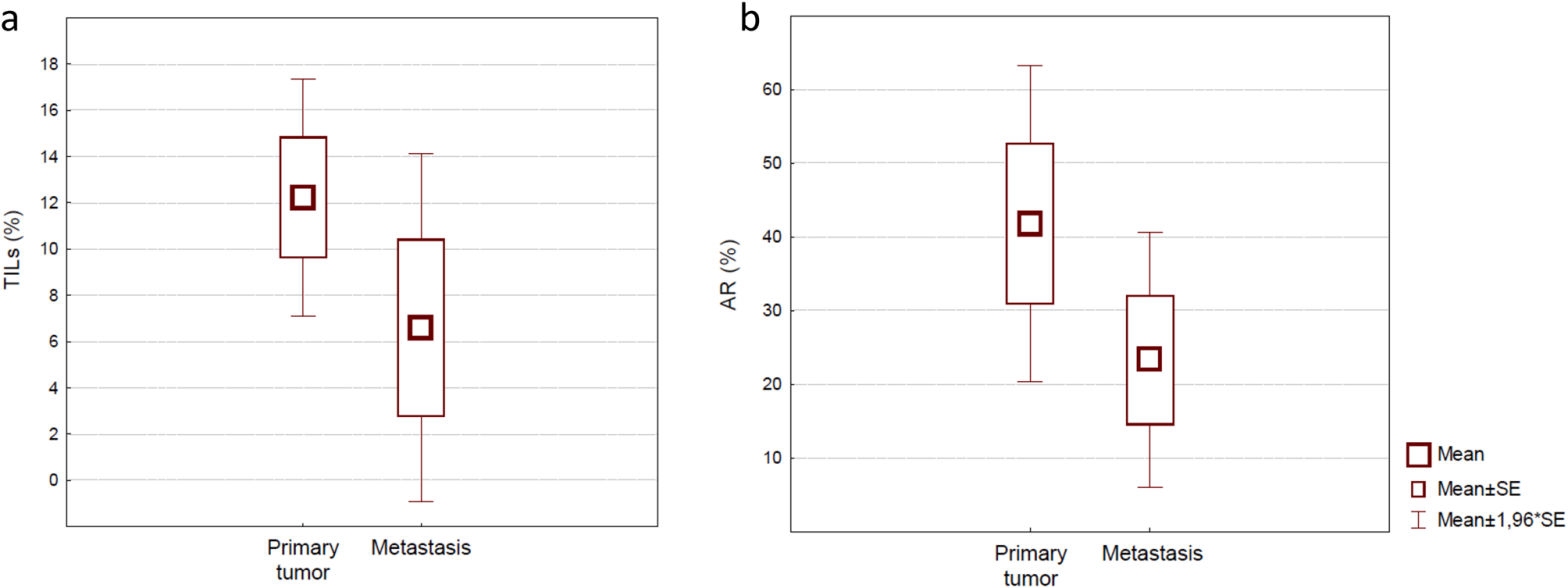
(**a**,**b**). Box and Whisker plot. Values of stromal TILs (**a**) and androgen receptor (**b**), shown as continue scale, differentially expressed between first diagnosis (primary tumor) and recurrence (distant metastases biopsies).

With a median follow-up of 6.16 years (range 0.89–13.12 years), we observed 38 (24%) distant recurrences, 4 (3%) contralateral breast cancers, and 2 (1%) ipsilateral breast cancers. Median time to metastasis was 15 months. Median number of metastatic sites was 3 (range 1–6). Thirteen out of 38 (34.2%) patients had visceral metastases, one (2.6%) patient recurred with bone-only disease and 27 (71.1%) developed central nervous system disease. For 17 (44.7%) patients, for whom biopsies of metastatic disease were available, we reviewed specimens and confirmed a TNBC phenotype. Our analysis showed that both TILs level and AR expression were lower within metastatic sites, in patients who developed distant metastases, compared to those found in the primary site, with a trend toward statistical significance (p = 0.08 for both; Fig. 3).

**Figure 4:**
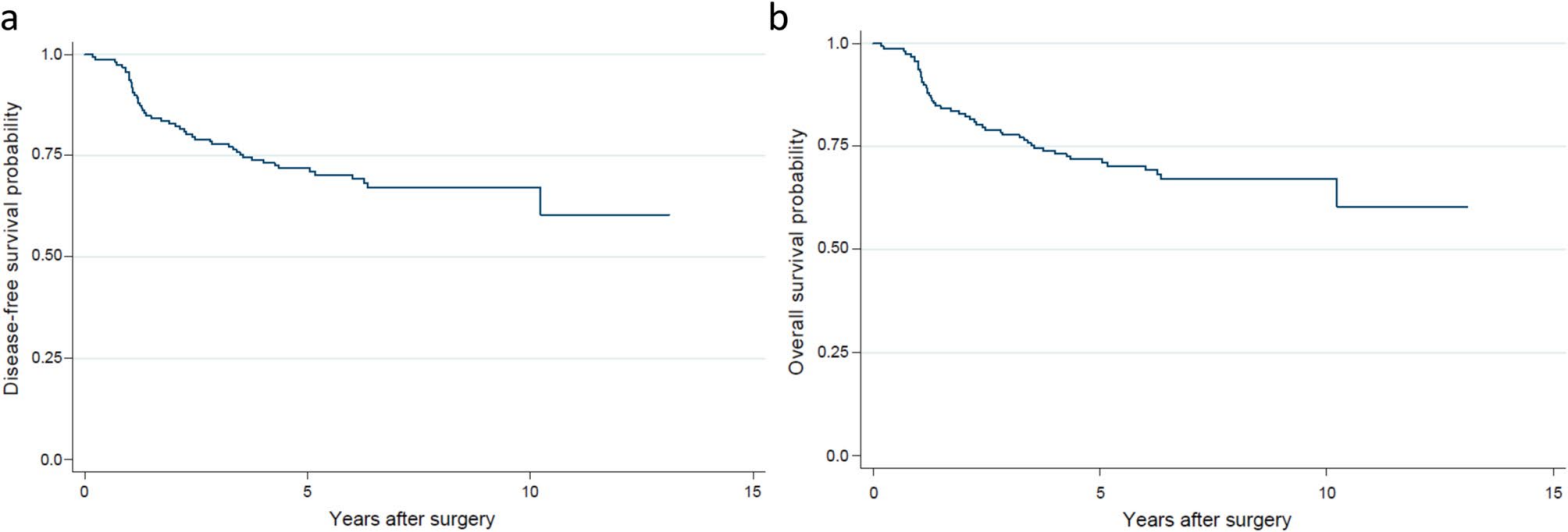
(**a**,**b**). Kaplan-Meyer survival estimate for DFS (**a**) and OS (**b**). *DFS* disease-free survival, *OS* overall survival.

With a high prevalence of small and node negative tumors and with a very low rate of disease-free and survival events, we were not able to identify any statistically significant association of TILs and AR with either DFS or OS (AR: p = 0.78 and p = 0.66 for DFS and OS, respectively; TILs: p = 0.94 and p = 0.46 for DFS and OS, respectively). Kaplan-Meyer curves for the entire population are shown in Fig. 4. Median DFS was 5.4 years (range 0.2–13.1 years) and median OS was 6.2 years (range, 0.9–15.1 years).

## Discussion and conclusion

In our monocentric, real life, retrospective case series, assessment of stromal TILs and AR was feasible and following international consensus guidelines^7^ we were able to correlate immune features with well-established pathological data. In line with literature data on high stromal TILs predicting benefit from chemotherapy, we confirmed that the higher stromal TILs scoring, the higher the aggressiveness of pathologic profile of the tumor. In our case-series, around half of the population showed AR positivity, in a mixed literature context, where values of AR positivity in TNBCs can range from 7 to 75%^15,18,28,32–34^. Interestingly, low Ki-67 came up to be the only pathologic feature significantly correlating with high AR expression, clearly linking lower biological aggressiveness with the luminal TNBC phenotype. These findings endorse AR as a biomarker to be used in clinical practice, together with Ki-67, to refine TNBC prognosis and help clinicians to better identify “low-proliferation” TNBC, for whom a tailored strategy, taking into account AR-targeted agents, may be proposed. Nevertheless, wide variability exists in the literature on AR + TNBC specific prognosis, with some studies reporting a correlation of the luminal TNBC subtype with increased lymph node metastasis, increased mortality, and poor disease free survival^21,35,36^. Besides, early phase trials testing different androgen-blockade strategies, have shown only limited clinical benefit rate (CBR) in AR + TNBCs (24-week CBR of 19% with bicalutamide^37^; 6-months CBR of 20% with abiraterone^38^ and 16-week CBR of 33% with enzalutamide^31^) and a phase III trial of paclitaxel plus enzalutamide versus placebo or enzalutamide monotherapy followed by paclitaxel was withdrawn (ClinicalTrials.gov identifier: NCT02929576). Moreover, when evaluating impact of AR in refining TNBC prognosis, probably a long-term follow-up, similar to the one routinely considered for luminal-like BCs, might be more suitable. Thus, many efforts still have to be made in defining AR+ TNBCs phenotype and clinical behavior, and possibly different biomarkers would need to be considered to refine luminal TNBCs prognosis, such as basal markers EGFR and CK 5/6, with EGFR−/AR + TNBCs exhibiting better clinical outcome compared to both EGFR+/AR+ and EGFR+/AR− disease^39^. Additionally, in line with the complexity of “luminal” TNBC, AR antagonists are being tested in combination with different targeted agents (such as CDK4/6 inhibitors and PI3K/AKT inhibitors) or immunotherapeutic strategies. The results of this research effort will hopefully help us identifying additional predictive biomarkers and understanding which proportion of AR+ TNBCs display a less aggressive behavior and fare prognostically better than other TNBCs.

In our case series most of the patients presented with stage I–II, with a low disease burden; specifically, around 35% of them had pT1N0 tumors, which harbor an intrinsic very good prognosis regardless of tumor biology^40^. In addition, due to the retrospective nature of the data and the relatively small sample size, we could not do an a-priori power analysis for interaction of AR and TILs with OS and DFS. These considerations are to be kept in mind when analyzing our Kaplan-Meyer curves, because we have to acknowledge that the effect of TILs and AR in modifying prognosis may be masked by the stage of presentation. Moreover, we think this finding to be specifically relevant nowadays, when due to widespread use of screening procedures, a very high percentage of tumors presents in stage I–II.

Our data on the extent of lymphocytic infiltration in metastasis biopsies are in line with recent literature, reporting a lower TILs score in metastatic tissue compared to the matched primary specimen^41–45^, with this trend being more frequently observed in more aggressive tumors, such as TNBC and HER2-enriched cases. Moreover, in our case series, biopsies of the metastatic site were taken at first appearance of metastatic disease, before the initiation of any chemotherapeutic line, thus no impairment of the immune system was induced by pre-treatment. These observation are of paramount interest because we now face the introduction in standard first-line therapy of immune check-point blockade for PD-L1 positive TNBC, where the amount of stromal reactive immune cells has been proved to be crucial for treatment benefit^46^. Thus further efforts have to be made to render the tumor microenvironment more sensitive to immune therapies; on this regard the recently published results of the TONIC trial indicate that short-term administration of chemotherapy, mainly doxorubicin and cisplatin, may induce a more favorable tumor microenvironment and increase the likelihood of response to immune check-point blockade in TNBC^47^.

Interestingly we found that age remained a statistically significant factor associated with both TILs and AR, with younger patients presenting with a more prominent stromal TILs infiltration and older patients expressing higher levels of AR. Age has been long considered a confounding factor in breast cancer prognosis definition, younger age being historically seen as a predictor for worse prognosis, independently of clinical and treatment-related variables^48–50^. Some authors have linked the more advanced and aggressive disease in young women to the increased potential for a delayed diagnosis, due to difficulties in detecting tumors because of the density of the mammary glands^51,52^. Nevertheless, it seems apparent that the prognostic impact of age is mainly guided by a more aggressive biological phenotype. Compared to older women, the young counterpart presents at diagnosis with higher percentage of ER and PgR negativity, vascular or lymphatic invasion and pathologic grade 3 tumors^53–56^. Thus, patient’s age has been endorsed to be considered as a prognostic factor by the National Comprehensive Cancer Network (NCCN), St. Gallen, and European Society of Medical Oncology (EMSO) guidelines^4,6,57^. Here, in line with the tight relationship between age and prognosis, we observed young women to present with a more aggressive phenotype than the older counterpart.

We also found a statistically significant association between high stromal TILs infiltration and low BMI, which is not surprising if we take into account data showing higher pCR rates after neo-adjuvant chemotherapy in lower mean BMI patients^58–62^. While in luminal/ER-positive BC subtypes numerous studies have identified an increased mortality risk in obese women^63–66^, BMI in TNBC is considered a confounding factor, as no direct relation has been established between BMI itself and recurrence-free survival (RFS) or OS in TNBC^67–69^. Here we propose a rationale for using BMI in refining TNBC prognosis in clinical practice, considering non-obese women to be at major risk for more aggressive, but chemo-sensitive, TN disease.

In conclusion, stromal TILs can be used to identify a more aggressive, but chemo-sensitive phenotype, mostly represented in younger women, while AR expression characterizes a less aggressive, slow-growing TNBC subtype, more common among older patients. Furthermore, we confirm that metastatic sites tend to be less rich in TILs, supporting a role for induction treatments prior to check-point blockade treatments. In our opinion, these findings are very interesting in managing daily clinical practice therapeutic decisions and prognosis speculations because, outside clinical trials, we face many pathological reports interpretation difficulties and refining prognosis with more biological parameters is of crucial importance.
